# Respiratory syncytial virus hospitalizations in US preterm infants after the 2014 change in immunoprophylaxis guidance by the American Academy of Pediatrics

**DOI:** 10.1038/s41372-020-0689-y

**Published:** 2020-06-04

**Authors:** Leonard R. Krilov, Evan J. Anderson

**Affiliations:** 1 Department of Pediatrics, NYU Winthrop Hospital and NYU Long Island School of Medicine, Mineola, NY USA; 2 Departments of Pediatrics and Medicine, Emory University School of Medicine, Atlanta, GA USA

**Keywords:** Viral infection, Viral infection

## Abstract

Palivizumab is the only licensed and effective immunoprophylaxis (IP) available to prevent respiratory syncytial virus (RSV) infection in high-risk infants including infants born at ≤35 weeks’ gestational age (wGA). In 2014, the American Academy of Pediatrics stopped recommending IP for otherwise healthy 29–34 wGA infants, stating that their risk of RSV hospitalization (RSVH) was similar to term infants. Recent studies have demonstrated a significant decline in IP use after 2014 that was accompanied by an increased risk of RSVH in 29–34 wGA infants vs term infants. Severity and healthcare utilization of RSVH were high among 29–34 wGA infants. In 2018, the National Perinatal Association developed guidelines advocating IP use in all ≤32 wGA infants and 32–35 wGA infants with additional risk factors. Risk factor predictive models can identify infants who are at risk for RSVH and promote cost-effective use of palivizumab until new methods of RSV prevention become available.

Respiratory syncytial virus (RSV) is the leading cause of lower respiratory tract infection (LRTI)-related hospitalizations, manifesting as bronchiolitis or pneumonia in infants aged <1 year in the United States [1–3]. The RSV season typically occurs annually from November through April in the Northern Hemisphere [4]. Nearly all children are infected at least once before the age of 2 years and about one-half are infected twice [5]. Hospitalization rates for RSV are about 16 times higher than those for influenza among children aged <1 year [6]. The incidence of RSV hospitalization (RSVH) is about 25.9 per 1000 infants at 1 month of age and 5.2 per 1000 among all infants younger than 2 years [7]. RSV is estimated to cause 100–500 deaths annually in children aged <5 years in the United States [8]. Infants who are at greatest risk for severe RSV disease include those born prematurely (≤35 weeks’ gestational age (wGA)), those with chronic lung disease of prematurity (CLDP, formerly known as bronchopulmonary dysplasia (BPD)), and infants with congenital heart disease (CHD) [9, 10].

## Burden of RSV in the pre-immunoprophylaxis era

Data are limited about the burden of RSV in preterm infants from the era before licensing of RSV immunoprophylaxis (IP). Boyce et al. conducted a retrospective analysis of children aged <3 years who were enrolled in the Tennessee Medicaid program from July 1989 through June 1993 to determine RSV-related hospitalization rates in high-risk population groups including early- and late-preterm infants (born at <36 wGA), infants with CLDP, and infants with CHD. Compared with full-term infants, preterm infants younger than 1 year had about a twofold greater risk of RSV-related hospitalization. The adjusted incidence rate ratio (RR) of RSVH for infants with CLDP and CHD aged <1 year was 10.7 and 2.8, respectively. All preterm infants were found to have a similar hospitalization rate irrespective of the degree of prematurity [9].

## Potential reasons for increased risk of RSV in preterm infants

Factors that may help explain the increased risk of RSVH in preterm infants relative to full-term infants include smaller lung volume, reduced alveolar surface area, reduced airway diameter, and increased air-space wall thickness [11, 12]. The narrow lower airway may be a major factor given the sloughing of airway epithelium that occurs with RSV resulting in inflammatory airway obstruction, atelectasis, hyperinflation, and increased risk of bacterial superinfection [13]. Alveolar development begins at ~30–32 wGA and alveoli are not fully developed in all infants until 36 wGA [11, 12]. In addition, preterm infants, particularly those born before 32 wGA, have minimal maternal antibody transfer, resulting in increased susceptibility to RSV and other infections, as more than 50% of maternal antibody transfer occurs after 34 wGA [13, 14]. Maternal immunoglobulin antibody transfer occurs rapidly, particularly from 33 to 36 wGA, to reach levels greater than those observed in the mother at term [15, 16]. After birth, anti-RSV maternal antibodies in healthy infants decrease to 73% at 1 month and 2% at 6 months of age, further increasing infant susceptibility to RSV [17].

## Prevention of RSVH with palivizumab—initial studies

The correlates of protection against RSV are incompletely understood. Despite this limitation, it is clear that RSV-specific antibodies play a crucial role in prevention of RSV-related disease [17, 18]. Subsequent studies of RSV hyperimmune globulin further suggested that passive immunization could prevent severe RSV in at-risk infants [19, 20]. This set the stage for the development of an RSV monoclonal antibody, palivizumab [4, 21–23].

The IMpact-RSV clinical trial was a randomized, double-blind, placebo-controlled trial conducted at 139 centers in the United States, the United Kingdom, and Canada from 1996 to 1997. It demonstrated the efficacy of palivizumab in protecting high-risk infants against severe RSV disease. A total of 1502 children who were (1) born prematurely (at ≤35 wGA) and were ≤6 months of age or (2) aged ≤24 months, born with CLDP, and required ongoing medical treatment were randomized to receive either palivizumab or placebo. A 55% reduction (*p* < 0.001) in RSV-related hospitalizations was observed in infants who received IP with palivizumab vs placebo. Palivizumab significantly reduced RSV-related hospitalization by 78% (*p* < 0.001) in premature infants without CLDP and 39% (*p* = 0.038) in premature infants with CLDP. Further subgroup analyses revealed an 80% reduction in hospitalizations in 32–35 wGA infants (*p* = 0.002) and a 47% reduction in hospitalizations in those <32 wGA (*p* = 0.003). Reported adverse events (AEs) were similar between the two groups and occurred in ≤3% of patients. The most frequent AEs were fever, nervousness, and injection site reactions that were mostly mild and transient [22]. In a subsequent study of infants with hemodynamically significant CHD, palivizumab reduced RSVH rates by 45% (*p* = 0.003) and the number of RSVH days with oxygen supplementation by 73% (*p* = 0.014), compared with placebo [23]. Another RSV monoclonal antibody, motavizumab, demonstrated noninferiority to palivizumab in reducing RSVH but this was not licensed by the Food and Drug Administration (FDA) [24].

Palivizumab is the only FDA-licensed intervention available for the prevention of serious LRTI caused by RSV in children [25]. It is indicated in high-risk infants with a history of premature birth (≤35 wGA) who are ≤6 months of age at the beginning of RSV season, those with CLDP or BPD who required medical treatment within the previous 6 months and who are aged ≤24 months at the beginning of RSV season, and those with hemodynamically significant CHD who are aged ≤24 months at the beginning of RSV season [4]. Although multiple vaccine candidates are undergoing clinical testing, it is likely that a safe and effective vaccine for RSV disease is years away from licensure [13, 21]. This is particularly true for preterm and younger infants who would have a longer window before vaccines might be extended down to their ages as additional safety studies will need to be performed in this population [26, 27].

## Prevention of RSVH with palivizumab—recent data

Palivizumab helps reduce severe RSV disease, however, some studies have suggested that it may increase non-RSV illnesses. Farber et al. retrospectively reviewed data from Texas and identified a significant decrease in RSVH with palivizumab IP (*p* = 0.04). They did note an increase in non-RSV bronchiolitis hospitalizations (*p* = 0.05) among infants born at 29–32 wGA [28], but they did not control for confounding by indication, which occurs when the indication for selecting a particular intervention also affects the result [29, 30]. In this case, the proportion of infants who received IP were significantly younger (*p* < 0.001) than non-palivizumab recipients and therefore were already at a higher risk of developing other infections, including non-RSV bronchiolitis (e.g., human metapneumovirus) [30, 31]. Similarly, a study by Achten et al. reported that prevention of RSV infection could increase the propensity for human rhinovirus infection in infants. Although such findings may be explained by the phenomenon of viral interference, the conclusions are mostly speculative and further data are needed [31]. In general, conclusions from nonrandomized, observational studies with potential confounding by indication should be evaluated carefully, and this potential bias should be identified and controlled for [30, 32]. Published, randomized, controlled trials of palivizumab vs placebo, by contrast, did not demonstrate an increase in non-RSV respiratory hospitalizations [22, 23].

A large, multicenter, test-negative case-control study established palivizumab effectiveness among preterm infants born at 29–35 wGA post licensure. The multiple logistic regression models used in the study calculated palivizumab effectiveness to be 74% (95% CI, 56–85%) among 29–35 wGA preterm infants aged ≤6 months without comorbid conditions including CHD and CLD. In addition, palivizumab reduced intensive care unit (ICU) admissions by 62% (95% CI, 35–78%) [29]. Moreover, a randomized, double-blind, placebo-controlled trial showed that palivizumab use reduced RSVH by 82% in infants born at 33–35 wGA during their first year of life. Benefit in prevention of subsequent wheezing episodes was also observed. Palivizumab significantly reduced the total wheezing days in the first year of life by 61% compared with placebo. These results not only implicate a role for RSV bronchiolitis in the development of early childhood wheezing, but also the potential for reducing early childhood wheezing with palivizumab IP [33].

## American Academy of Pediatrics 2014 guidance for IP use in preterm infants

Since FDA approval of palivizumab in 1998, the American Academy of Pediatrics (AAP) Committee on Infectious Diseases has serially revised recommendations for the use of RSV IP [4, 34]. From 2009 until 2014, the AAP recommended RSV IP for all preterm infants born at <32 wGA and at 32 to <35 wGA and <3 months of chronological age (CA) at the start of the RSV season with at least one additional risk factor (e.g., childcare attendance, those with at least one sibling or other child <5 years that lives permanently in the same household) (Table 1) [35]. In 2014, the AAP reviewed epidemiologic data from the palivizumab IP era and concluded that ≥29 wGA infants have RSVH rates similar to those of full-term infants [36]. They then stopped recommending RSV IP for infants born at ≥29 wGA without comorbidities such as CLDP and CHD [34]. However, as it has been observed, studies used to develop the guidance were (1) not sufficiently powered to determine RSVH incidence in premature infants, (2) only representative of three counties within the United States that may not be generalizable, or (3) performed during the palivizumab era, which may explain why RSVH rates in preterm infants were similar to the rates of full-term infants [32, 37]. Overall, the stringent guidance seems to be influenced by misguided interpretation of information that led to the restriction of IP use in these high-risk infants [32, 37].

**Table 1:** Guidance for RSV IP in preterm infants^a^.

Preterm infants	AAP 2009 guidance [35]	AAP 2014 guidance [34]	NPA 2018 guidelines [53]
<29 wGA	<12 months of age at season start	<12 months of age at season start	<12 months of age at season start
29–31 wGA	<6 months of age at season start	Not recommended	<6 months of age at season start
32–35 wGA	<3 months with identified risk factors in those 32 to <35 wGA^b^	Not recommended	<6 months of age at season start (includes 35 wGA infants) with significant provider-identified risk factors^c^

*AAP* American Academy of Pediatrics, *IP* immunoprophylaxis, *NPA* National Perinatal Association, *RSV* respiratory syncytial virus, *wGA* weeks’ gestational age.

^a^Palivizumab is approved for all ≤35 wGA infants aged ≤6 months at the beginning of RSV season [4].

^b^Childcare attendance or one or more siblings <5 years of age.

^c^Childcare attendance, one or more school-aged siblings, twin or greater multiple gestation, young chronological age at the start of RSV season, and parental smoking.

Since that time, multiple studies have assessed the impact of the change in the 2014 guidance on hospitalization risk and rates, severity, and costs in 29–34 wGA infants. This recent evidence includes studies based on large patient databases, as well as multicenter and single-center studies (Table 2). This review will discuss changes in RSV disease epidemiology and burden after the AAP guidance changes.

**Table 2:** Evidence-based studies since 2014.

Study (Season)	Study description	Age group	Key outcomes
Anderson et al.; SENTINEL1 (2014–2016) [44, 45]	Multicenter, noninterventional, observational cohort	29–35 wGA <12 months CA	• RSVH was severe and often required ICU admission and IMV in most preterm infants who did not receive IP • Infants of earlier GA and younger CA were associated with higher disease severity (ICU admission and need for IMV) and hospital charges
Kong et al. (2013–2014 vs 2014–2015) [38]	Truven Commercial and Medicaid databases	29–34 wGA <6 months CA	• RSV IP use decreased significantly • Rate ratio of RSVH in preterm infants vs term infants increased
Goldstein et al. (2012–2014 vs 2014–2016) [39]	Truven Commercial and Medicaid databases	29–34 wGA <6 months CA	• RSV IP use decreased significantly • Rate ratio of RSVH in preterm infants vs term infants increased
Rajah et al. (2013–2014 vs 2014–2015) [47]	Single-center, retrospective study	29–34 wGA <12 months CA	• RSVH, severity, and hospital charges significantly increased in preterm infants born at 29–34 wGA
Blake et al. (2012–2014 vs 2014–2016) [48]	Single-center, retrospective cohort	29–32 wGA <12 months CA	• RSV IP use decreased significantly • RSVH increased significantly in preterm infants born at 29–32 wGA
Farber et al. (2012–2014 vs 2014–2015) [40]	Retrospective study from Texas	29–32 wGA <6 months CA	• No significant change in RSVH
Grindeland et al. (2012–2014 vs 2014–2015) [42]	Single-center, retrospective study	<2 years CA	• RSV IP use decreased significantly • No significant change in RSVH
Zembles et al. (2012–2014 vs 2014–2017) [46]	Single-center, retrospective study	29–35 wGA <12 months CA	• No significant change in RSVH or morbidity except for duration of hospitalization

*CA* chronological age, *GA* gestational age, *ICU* intensive care unit, *IMV* invasive mechanical ventilation, *IP* immunoprophylaxis, *RSV* respiratory syncytial virus, *RSVH* respiratory syncytial virus hospitalization, *wGA* weeks’ gestational age.

## Outpatient palivizumab use decreased after 2014 guidance

**Fig. 1:**
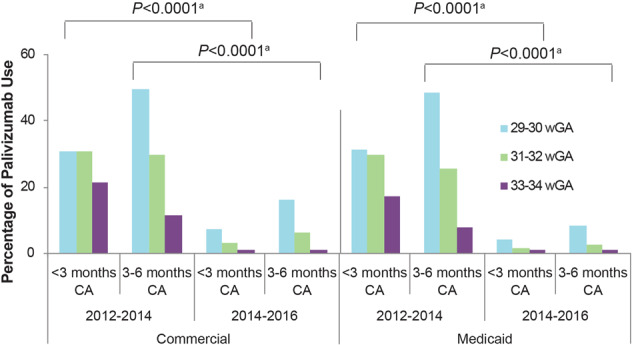
Palivizumab use decreased after 2014 [39]. CA, chronological age; wGA, weeks’ gestational age. ^a^*p* < 0.0001 for all preterm infants, regardless of gestational age, between the 2014–2016 vs 2012–2014 seasons. Republished with permission of *Am J Perinatol*, from Goldstein M, et al. 35(14) 2018 permission conveyed through Copyright Clearance Center, Inc.

After the release of the 2014 guidance, evidence suggests that use of palivizumab IP decreased substantially. Two analyses based on the Truven Health MarketScan Commercial and Medicaid health insurance administrative claims databases compared RSV IP use among preterm infants in the seasons before and after the guidance change. Kong et al. compared the 2014–2015 season with the 2013–2014 season and included about 1.2 and 1.4 million commercially and Medicaid-insured infants, respectively. Between the 2013–2014 and 2014–2015 seasons, the proportion of 29–34 wGA preterm infants receiving at least one dose of outpatient RSV IP decreased (*p* < 0.01) among commercially and Medicaid-insured infants. These declines ranged from 45 to 95% among preterm groups. The decline of outpatient IP use was highest among infants born at 29–30 wGA and <6 months CA [38]. Similarly, Goldstein et al. compared outpatient RSV IP use in the two seasons before (2012–2013 and 2013–2014) and two seasons after (2014–2015 and 2015–2016) the guidance change. The study analyzed commercially insured (33,667 preterm infants born at 29–34 wGA and 668,619 term infants) and Medicaid-insured infants (51,439 preterm infants born at 29–34 wGA and 908,594 term infants). The proportion of preterm infants receiving RSV IP declined by ≥74% for all groups analyzed (*p* < 0.0001, both commercial and Medicaid-insured infants) in 2014–2016 vs 2012–2014 (Fig. 1) [39].

## Impact of the 2014 changes in AAP guidance on RSVH burden in geographically limited studies

Data from geographically limited studies have been mixed in demonstrating the impact of the guidance change on the burden of RSVH. Three studies did not show an increase in RSVH following the guidance change in 2014 for RSV IP. Farber et al. examined RSVH rates during the 2012–2014 seasons in otherwise healthy infants born at 29–36 wGA using pooled health plan claims data from nine Texas Medicaid managed care programs. Administration of ≥1 dose of palivizumab was associated with a significant decrease in RSVH (*p* = 0.04) among infants born at 29–32 wGA; however, there was no significant change in RSVH among infants born at 33–36 wGA (*p* = 0.7) [28]. Important limitations include that <50% of the infants born at 29–32 wGA had received ≤50% of the indicated doses and the proportion of RSV IP use among infants 33–36 wGA was not reported. In addition, infants who received IP were significantly younger (*p* < 0.001) than non-palivizumab recipients [30]. Farber et al. performed a separate analysis using pooled data from the eight health plans that had 3-year data (2012–2015, *n* = 2031) and reported no statistically significant difference in the RSVH rates among preterm infants born at 29–32 wGA after 2014 (5.41% in 2014–2015 compared with 3.06% in 2013–2014). Based on the results, the authors concluded that the year-to-year changes in RSVH among infants born at 29–32 wGA were not affected by the 2014 guidance change [40]. However, at the time of this study, Texas Medicaid had not adopted the 2014 AAP guidance to restrict IP use for infants 29–32 wGA, and the proportion of infants who received palivizumab was not reported [34, 41].

In a retrospective analysis conducted in North Dakota, Grindeland et al. compared the incidence of RSVH among infants aged <2 years in the seasons before (2012–2014, *n* = 23,085) and after (2014–2015, *n* = 12,107) the guidance change. Palivizumab use decreased significantly (*p* < 0.0001) in 2014–2015 compared with 2012–2014, but this was not associated with an overall increase in the incidence of RSVH (*p* = 0.6) [42]. However, this study did not specifically examine the IP-indicated high-risk population including preterm infants born at <35 wGA and did not have the statistical power to detect clinically plausible differences [42, 43]. Had the analysis been stratified by CA, significant differences in RSVH may have been observed [44, 45].

In a single-center, retrospective study, Zembles et al. analyzed 664 RSVH that occurred in the 2012–2017 seasons and found no significant change in RSVH among infants following the AAP guidance change over three subsequent seasons (2014–2017). Nonetheless, the authors did observe an increase in the number of RSVH in the first season (2014–2015) after the guidance change. There was no significant change in the proportion of infants who required ICU admission or mechanical ventilation (MV) in the 2014–2017 vs 2012–2014 seasons; however, duration of hospitalization that occurred in the first year of life was significantly longer in 2014–2017 than in 2012–2014 (*p* = 0.02) [46]. Of note, when RSVH were compared before and after 2014 among preterm infants born at 29–34 wGA, the proportion of all RSVH almost doubled in 2014–2017 (17.2%) compared with 2012–2014 (9.7%; *p* = 0.0047, Chi-square test) (unpublished data).

By contrast, other studies have identified a substantial impact of the AAP guidance changes on RSVH. Rajah et al. at Nationwide Children’s Hospital in Ohio reported an increase in RSVH in the 2014–2015 season compared with the 2013–2014 season among preterm infants born at 29–34 wGA. The proportion of preterm infants aged <6 months with RSVH was significantly higher in 2014–2015 (7.1%) than in 2013–2014 (3.5%; *p* = 0.01) [47]. In addition, Blake et al. retrospectively analyzed 173 infants and found that RSVH was significantly higher (*p* = 0.04) among infants born at 29–32 wGA in 2014–2016 vs 2012–2014. This also coincided with significant decreases in palivizumab prescriptions beginning in 2014 (*p* = 0.01) [48]. However, a major setback of these single-center studies is the lack of a denominator to account for seasonal variation; other large studies have taken this into consideration and are discussed below [40, 47, 48].

## Impact of the 2014 changes in AAP guidance on RSVH burden using data from large databases

Two database studies of large numbers of US infants analyzed the risk of RSVH among preterm infants born at 29–34 wGA relative to full-term infants following decreased IP use after 2014. In the Kong et al. study, RSVH rates in the 2014–2015 vs 2013–2014 seasons were 2.65 (*p* = 0.02) and 1.41 (*p* = 0.03) times higher among infants 29–34 wGA and <3 months CA in commercial and Medicaid insurance groups, respectively. The average RSVH rates in preterm infants <3 months CA were also significantly higher in 2014–2015 when compared with the combined 2010–2014 seasons. In contrast, RSVH rates among full-term infants were similar in the 2013–2014 and 2014–2015 seasons [38].

**Fig. 2:**
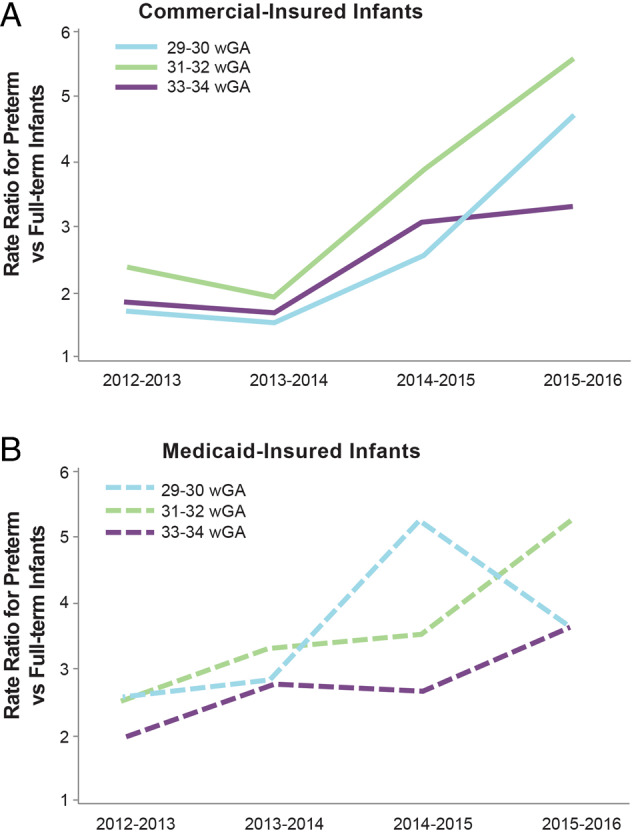
Hospitalization risk increased after 2014 in 29–34 wGA preterm infants vs term infants aged <6 months. **a** Commercial-insured infants; **b** Medicaid-insured infants [39]. RSV, respiratory syncytial virus; wGA, weeks’ gestational age. Republished with permission of *Am J Perinatol*, from Goldstein M, et al. 35(14) 2018 permission conveyed through Copyright Clearance Center, Inc.

Goldstein et al. reported that the risk of RSVH was two times greater in commercially insured (*p* < 0.0001) and 1.5 times greater in Medicaid-insured (*p* < 0.0001) preterm infants 29–34 wGA and <6 months CA compared with term infants in the 2014–2016 vs 2012–2014 seasons. The seasonal RSVH RRs in preterm infants vs term infants in 2012–2014 ranged from 1.6 to 3.4; these increased to 2.6–5.6 in the 2014–2016 seasons (Fig. 2). The risk of RSVH for preterm vs term infants increased after 2014 and the highest RSVH rates were found in infants of earlier GA (29–30 wGA) and younger CA (aged <3 months) [39]. These data showed that the RSVH risk increased among preterm infants relative to term infants concurrent with decreases in IP use [38, 39].

## RSVH severity in 29–35 wGA preterm infants (2014–2016 seasons)

**Fig. 3:**
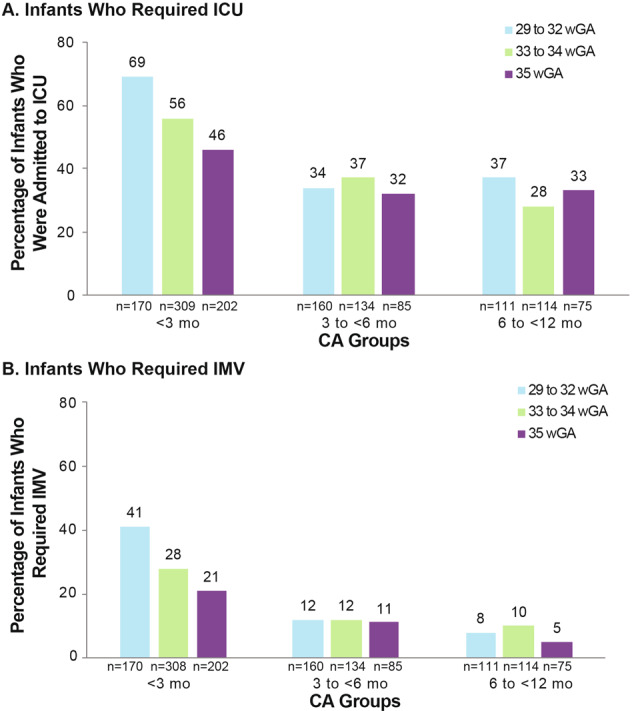
Percentage of preterm infants who required ICU and IMV in 2014–2016 seasons. **a** Infants who required ICU; **b** Infants who required IMV [44]. ICU, intensive care unit; IMV, invasive mechanical ventilation; wGA, weeks’ gestational age. Republished with permission of *Am J Perinatol*, from Anderson EJ, et al. doi: 10.1055/s-0039-1681014 2019 permission conveyed through Copyright Clearance Center, Inc.

Preterm infants are vulnerable to severe RSV infections that require hospitalization and intensive care, and there may have been an increase in severity in these infants after palivizumab IP recommendations changed. SENTINEL1, a retrospective and prospective observational analysis, was the largest study to characterize hospitalization among preterm infants born at 29–35 wGA with laboratory-confirmed community-acquired RSV disease who did not receive RSV IP. The study analyzed two seasons (2014–2015 and 2015–2016; *n* = 1378) and was conducted across more than 40 US centers [44]. SENTINEL1 analyzed infants aged <12 months and provided a robust US-specific estimate of the morbidity associated with RSVH. The vast majority of RSVH (78%), ICU admission (84%), and invasive mechanical ventilation (IMV) (91%) occurred among infants within 6 months of birth. When data from both seasons were combined, earlier GA combined with younger CA was associated with higher rates of ICU admission and the need for IMV (Fig. 3). The mean hospital length of stay for the combined 2014–2016 seasons was 10, 9, and 7 days for infants born at 29–32 wGA, 33–34 wGA, and 35 wGA, respectively. The duration of hospitalization and the duration of ICU stay were longer among infants of younger CA. Results were corroborated by multivariate logistic regression analyses, which confirmed that increased prematurity at delivery and younger CA were consistently associated with ICU admission and need for IMV [44]. Some limitations of these large database studies include potential undercoding of RSVH due to lack of confirmatory diagnosis of RSV, underestimation of RSV IP use as inpatient palivizumab receipt was not recorded, and small power in earlier gestational age groups [38, 39]. Recently, Arriola et al. estimated a total of 1554 RSVH in four US locations among children aged <2 years in the 2014–2015 RSV season; 27% and 6% of these hospitalizations required ICU admission and MV, respectively. Three of the five children who died were 33–35 wGA infants and four of the deaths were in children aged ≤6 months. Although palivizumab receipt was not recorded in these infants, the data highlight the potential risk of RSVH mortality associated with moderate-late-preterm infants [49].

Similar to the SENTINEL1 study, RSVH was more severe among preterm infants aged <3 months in the Rajah et al. study in the 2014–2015 season compared with the 2013–2014 season. Admission to the ICU among infants aged <3 months was significantly higher in 2014–2015 (68.4%) than in 2013–2014 (30.0%; *p* = 0.04). The need for IMV and duration of hospitalization in infants aged <3 months also increased significantly in 2014–2015 vs 2013–2014 (*p* = 0.04 for both). The median duration of RSVH was about 4.5 times greater in the 2014–2015 season compared with the 2013–2014 season among infants aged <3 months and 3 to <6 months [47].

## Costs associated with RSVH of 29–35 wGA preterm infants

**Fig. 4:**
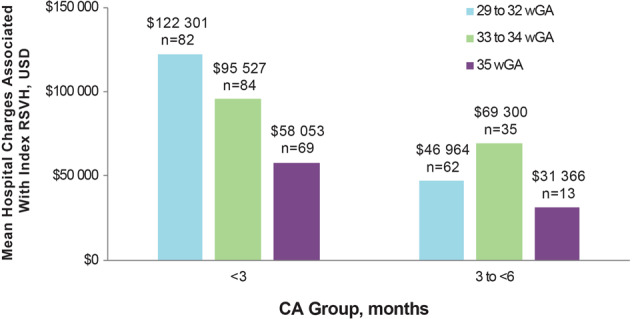
Mean hospital charges for infants 29–35 wGA hospitalized with RSV infection by GA and CA for combined seasons. [44] CA, chronological age; GA, gestational age; RSV, respiratory syncytial virus; RSVH, respiratory syncytial virus hospitalization; USD, United States dollars; wGA, weeks’ gestational age. Republished with permission of *Am J Perinatol*, from Anderson EJ, et al. doi: 10.1055/s-0039-1681014 2019 permission conveyed through Copyright Clearance Center, Inc.

RSV infections that require hospitalization, intensive care, and IMV are expensive. For the combined 2014–2016 seasons in the SENTINEL1 study, the mean hospital charges for preterm infants born at 29–32 wGA and <3 months CA were $122,301 (Fig. 4) [44]. Goldstein et al. reported that in the 2014–2016 seasons, the mean costs of RSVH were more than two times greater among preterm infants aged <3 months than among term infants for both commercial ($41,104 vs $17,597) and Medicaid-insured ($24,049 vs $10,897) infants. Similar to the SENTINEL1 study, higher costs were associated with RSVH occurring in preterm infants born at 29–34 wGA of younger CA [39].

Rajah et al. also compared RSVH-associated hospital charges in the seasons before and after 2014. The median charges for RSVH were significantly higher in the 2014–2015 vs 2013–2014 seasons for preterm infants born at 29–34 wGA with comorbidities ($31,339 vs $19,947; *p* = 0.02) and without comorbidities ($30,662 vs $19,686; *p* = 0.03) [47].

Blake et al. estimated that the direct costs associated with RSVH per day were $2909 with prior IP use and $32,238 without IP use, and that 20 infants at a cost of $90,000 would need to be treated with palivizumab in order to prevent one RSVH with a projected cost of $29,000. The authors acknowledged that the small sample size expected from a single institution, lack of complete financial and clinical information, and possible interference due to unidentified prior IP use could all limit the generalization of the results [48]. Whether reduction in hospital costs outweighs the high acquisition cost of IP at the current population level needs to be studied. As palivizumab is generally well-tolerated with rare serious adverse events (SAE) associated with its use both in the pivotal trials as well as in real-world surveillance, the potential for SAE expansion with increased use should not be a concern but warrants further investigation [22, 23, 50]. Overall, results from several large database and independent single-center studies confirmed that there was a substantial cost associated with RSVH in preterm infants born at 29–34 wGA.

## Risk factor predictive model in moderate-late-preterm infants

Compared with term infants, moderate-late-preterm infants (born at 32–35 wGA) have a higher risk of developing severe RSV disease. The prospective RSV Respiratory Events Among Preterm Infants Outcomes and Risk Tracking (REPORT) study conducted in the United States during the 2009–2011 seasons reported that ~1 RSVH occurred per 20 infants born at 32–35 wGA and <6 months CA at the start of the RSV season who did not receive IP. Risk of developing severe RSV disease was highest among infants born at 32–35 wGA who attended day care and those with preschool-aged siblings. This is consistent with the 2012 AAP guidance that suggested these environmental risk factors identify a group of high-risk 32–35 wGA infants. Other associated risk factors for RSVH were exposure to smoking and CA <3 months. Of note, ~75% of severe RSV disease occurred among infants aged <6 months with risk factors [51]. A pooled analysis of seven prospective observational studies (including the REPORT study) conducted from 2000 to 2014 among infants born at 33–35 wGA reported an RSVH incidence rate of 3.4%, of which 22.2% and 12.7% required ICU admission and MV, respectively [52]. These results also underscore the substantial severe RSV-associated burden in moderate-late-preterm infants who do not receive IP.

Recently, Blanken et al. developed a simple risk factor analyzing tool from a pooled analysis of six large, multicenter, prospective, observational studies in the Northern Hemisphere evaluating 32–35 wGA infants. Logistic regression models identified three risk factors with high accuracy (*p* ≤ 0.001) that predicted RSVH in moderate-late-preterm infants. These include (1) birth between 3 months before and 2 months after RSV season start date, (2) exposure to smoking during pregnancy or after birth, and (3) multiple siblings and/or day care attendance [25].

**Fig. 5:**
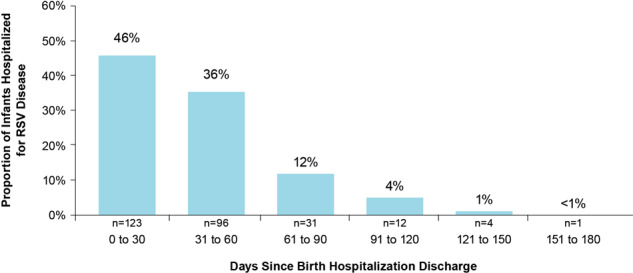
Timing between in-season birth discharge and RSVH. RSV, respiratory syncytial virus; RSVH, respiratory syncytial virus hospitalization. Unpublished data.

Important data have recently been generated regarding timing of RSVH after birth hospitalization discharge. Anderson et al. estimated that two-thirds (73%) of RSVH in preterm infants 29–35 wGA occurred among infants who were discharged from their birth hospitalization during the 6-month period from September to February. Among those infants discharged from their birth hospitalization between November and March, ~46% and 82% of RSVH occurred within the first 30 and 60 days after discharge, respectively (Fig. 5). These findings emphasize the increased risk of RSV infection associated with younger CA and the timing of birth hospitalization discharge in relation to RSV season [44]. Region-specific and consistently identified risk factors for RSVH and the clinical data available on the timing of RSVH and birth hospitalization discharge may help identify infants at the highest risk of RSVH and promote cost-effective use of palivizumab [25, 44]. Additional studies are needed to investigate whether initiating RSV IP in high-risk infants at the time of birth hospitalization discharge could prevent sufficient RSVH to change the overall cost equation of RSV IP.

## National Perinatal Association 2018 guidelines for RSV IP in preterm infants

Since 2014, guidance changes have resulted in declines in palivizumab use with subsequent increases in RSVH risk, RSVH severity, and cost. Based on the growing evidence of RSV disease burden and severity since 2014, the National Perinatal Association issued RSV IP guidelines in 2018. They have recommended expanding RSV IP to include infants born at 29 to <32 wGA and aged <6 months at the start of RSV season. For 32–35 wGA preterm infants, IP is recommended after special consideration and assessment of factors that may increase the risk of RSVH and morbidity (e.g., childcare attendance, school-aged siblings, twin or greater multiple gestation, young CA at the start of RSV season, and parental smoking) (Table 1) [53].

## Conclusions

Recent evidence indicates the substantial burden associated with RSVH among preterm infants 29–35 wGA without comorbidities. However, in 2019, the AAP COID reaffirmed the 2014 guidance to exclude 29–34 wGA preterm infants, without providing specific reasons [28]. Until vaccines or new monoclonal antibodies become available, IP with palivizumab in high-risk preterm infants born at <35 wGA, especially shortly after birth hospitalization discharge, could reduce the significant morbidity and economic burden caused by RSVH.
